# Ecological Niche Modeling to Estimate the Distribution of Japanese Encephalitis Virus in Asia

**DOI:** 10.1371/journal.pntd.0001678

**Published:** 2012-06-19

**Authors:** Robin H. Miller, Penny Masuoka, Terry A. Klein, Heung-Chul Kim, Todd Somer, John Grieco

**Affiliations:** 1 Department of Preventive Medicine and Biometrics, Uniformed Services University of the Health Sciences, Bethesda, Maryland, United States of America; 2 65th Medical Brigade/USAMEDDACKorea, Unit 15281, Force Health Protection and Preventive Medicine, Yongsan Army Garrison, Seoul, Republic of Korea; 3 168th Multifunctional Medical Battalion, 65th Medical Brigade, Unit 15247, 5th Medical Detachment, Yongsan Army Garrison, Seoul, Republic of Korea; 4 NST, National Geospatial-Intelligence Agency, Fort Detrick, Maryland, United States of America; NASA Goddard Space Flight Center, United States of America

## Abstract

**Background:**

*Culex tritaeniorhynchus* is the primary vector of Japanese encephalitis virus (JEV), a leading cause of encephalitis in Asia. JEV is transmitted in an enzootic cycle involving large wading birds as the reservoirs and swine as amplifying hosts. The development of a JEV vaccine reduced the number of JE cases in regions with comprehensive childhood vaccination programs, such as in Japan and the Republic of Korea. However, the lack of vaccine programs or insufficient coverage of populations in other endemic countries leaves many people susceptible to JEV. The aim of this study was to predict the distribution of *Culex tritaeniorhynchus* using ecological niche modeling.

**Methods/Principal Findings:**

An ecological niche model was constructed using the Maxent program to map the areas with suitable environmental conditions for the *Cx. tritaeniorhynchus* vector. Program input consisted of environmental data (temperature, elevation, rainfall) and known locations of vector presence resulting from an extensive literature search and records from MosquitoMap. The statistically significant Maxent model of the estimated probability of *Cx. tritaeniorhynchus* presence showed that the mean temperatures of the wettest quarter had the greatest impact on the model. Further, the majority of human Japanese encephalitis (JE) cases were located in regions with higher estimated probability of *Cx. tritaeniorhynchus* presence.

**Conclusions/Significance:**

Our ecological niche model of the estimated probability of *Cx. tritaeniorhynchus* presence provides a framework for better allocation of vector control resources, particularly in locations where JEV vaccinations are unavailable. Furthermore, this model provides estimates of vector probability that could improve vector surveillance programs and JE control efforts.

## Author Summary

Japanese encephalitis virus (JEV) is transmitted predominately by the mosquito, *Culex tritaeniorhynchus*. The primary reservoirs of the virus are wading birds, with swine serving as amplifying hosts. Despite the development of a JEV vaccine, people remain unvaccinated in endemic countries and are susceptible to JEV infection. The distribution of the JEV vector(s) provides essential information for preventive measures. This study used an ecological niche modeling program to predict the distribution of *Cx. tritaeniorhynchus* based on collection records and environmental maps (climate, land cover, and elevation). The model showed that the mean temperatures of the wettest quarter had the greatest impact on the model. Of the 25 countries endemic for Japanese encephalitis (JE) endemic countries, seven possessed greater than 50% land area with an estimated high probability of *Cx. tritaeniorhynchus* presence. Our model provides a useful tool for JEV surveillance programs that focus on vector control strategies.

## Introduction

**Figure 1:**
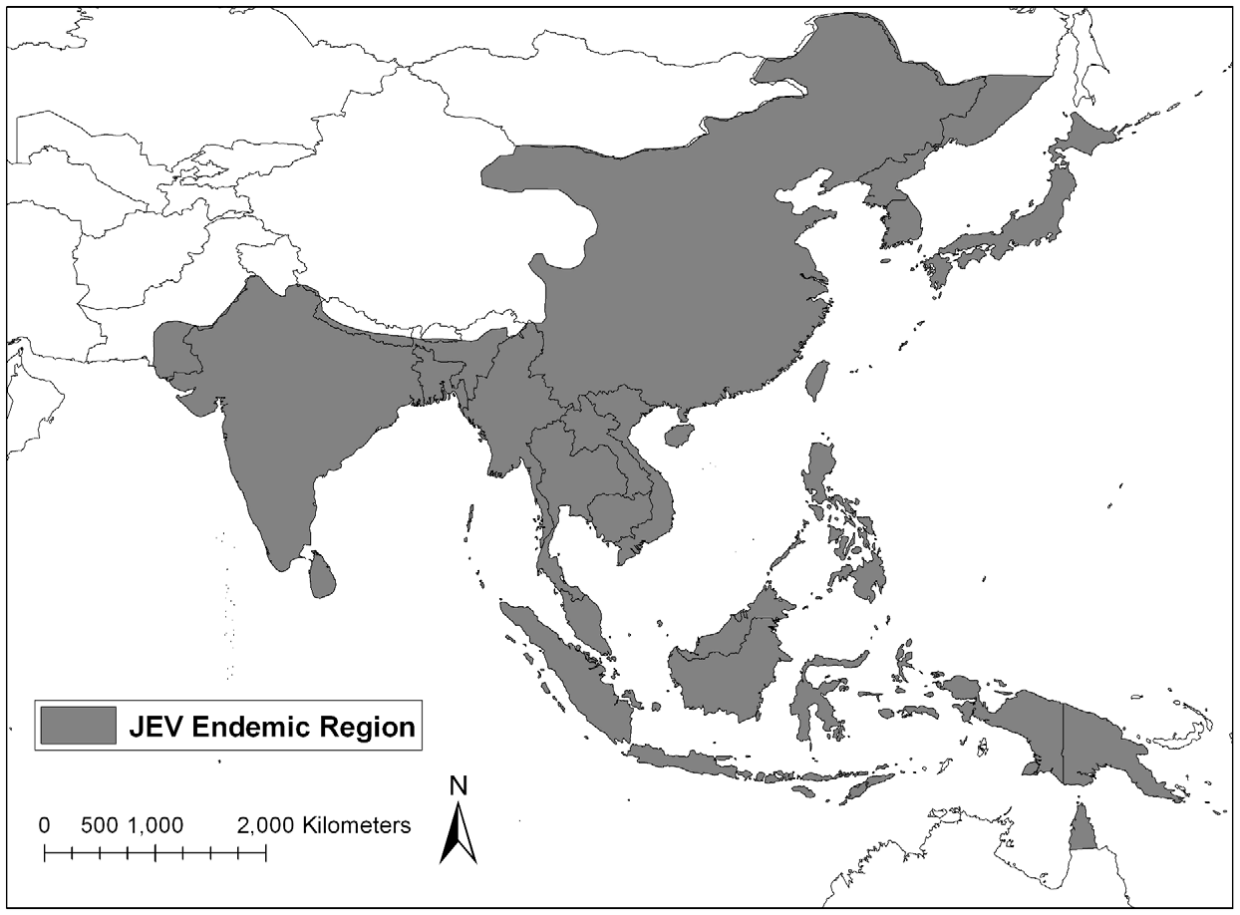
Japanese encephalitis virus endemic area. Map adapted from CDC.

Japanese encephalitis virus (JEV), the causative agent of Japanese encephalitis (JE), is an arbovirus that belongs to the family *Flaviviridae* and is endemic to Southeast and Northeast Asia, the Pacific Islands, and northern Australia (Figure 1) [1]. The primary vector of JEV is *Culex tritaeniorhynchus* Giles, but other *Culex* species (e.g., *Culex annulirostris*, *Culex vishnui* Theobald, *Culex bitaeniorhynchus* Giles, and *Culex pipiens* Linnaeus) have also been implicated as important viral transmitters [2], [3], [4], [5]. The larval habitat of *Cx. tritaeniorhynchus* is primarily low lying flooded areas containing grasses and flooded rice paddies, but this species can also be found in urban environments in close proximity to human populations [6]. Within the past 40 years, rice agriculture in JEV endemic countries has increased by 20%, thereby expanding *Cx. tritaeniorhynchus* habitat and increasing human risk of exposure to vector populations [7].

Swine, including domestic and feral pigs, serve as amplifying hosts of JEV in endemic areas. The proximity of human populations to pig farms, sties or feral pig populations increases the risks of JEV exposure [8], [9]. Ardeid birds (large wading birds) are an important JEV reservoir and can spread JEV to new regions through their northern migration to breeding and feeding grounds in the spring and southern return in the fall [3]. Additional animals have been identified as host species for JEV, including domesticated animals (chickens, goats, cows, and dogs), as well as bats, flying foxes, ducks, snakes and frogs. However, these are considered dead-end hosts as they infrequently develop sufficient viremias to infect mosquito vectors [10], [11], [12], [13].

Despite the introduction of an effective vaccine to the public in the mid-1900s, JEV remains the leading cause of viral encephalitis globally [14]. Comprehensive vaccination programs in Japan, Republic of Korea (ROK), Brunei, Australia, and Malaysia have significantly reduced the number of human cases [15]. Rare occurrences of neurological complications associated with the mouse-brain derived JEV vaccine interrupted vaccination programs in some regions, initiating concerns of the reemergence of JEV in an unvaccinated and non-immune population [16], [17]. The prevalence of JE is higher in countries with lower socioeconomic status, when compared to more affluent neighboring countries, indicating the importance of economic and social stability as additional risk factors that impact the transmission and prevalence of JE in non-immune populations [15].

Recent developments in the field of ecological niche modeling and the development of global environmental data sets have resulted in the ability to predict the distribution of vector populations that directly relate to transmission of viruses, parasites, and fungal pathogens and impact on animal and human health. Modeling to estimate the distribution of disease vectors provides useful information in disease-endemic areas, in addition to predicting how anthropogenic changes to the environment will affect disease presence [18], [19], [20], [21].

In the current study, the Maxent ecological niche modeling program was utilized to model the distribution of the primary vector of JEV, *Cx. tritaeniorhynchus* [22]. The resulting vector habitat suitability map was compared to the reported locations of JE human cases and the current status of established JE vaccination programs by country. Our ecological niche model can be used by public health officials and government agencies in endemic regions to guide implementation of comprehensive vaccination programs, vector control strategies, and public health awareness campaigns.

## Methods

### *Culex tritaeniorhynchus* Data Collection

Geographical coordinates of known *Cx. tritaeniorhynchus* records were identified by performing a literature search in PubMed for all previous field collection studies. When exact geographical coordinates were not provided, locations were approximated by searching for the given city, town, or village using Google Earth software. Further geographical data points for the distribution of *Cx. tritaeniorhynchus* were obtained through MosquitoMap (http://www.mosquitomap.org/), a database of spatial data points of mosquitoes that is maintained by the Walter Reed Biosystematics Unit, Smithsonian Support Center, Silver Hill, MD. Additional *Cx. tritaeniorhynchus* collection data were obtained from Force Health Protection and Preventive Medicine, 65^th^ Medical Brigade, Yongsan Army Garrison, ROK.

In previous modeling work in the ROK [20], we found that a large number of collection records in a limited geographical area biased the model. As a result of the large number of collection sites for the ROK (96 unique locations), we reduced the number of records to 23 by deleting all but one randomly selected record per administrative district.

### Identification of Japanese Encephalitis (JE) Human Cases

**Figure 2:**
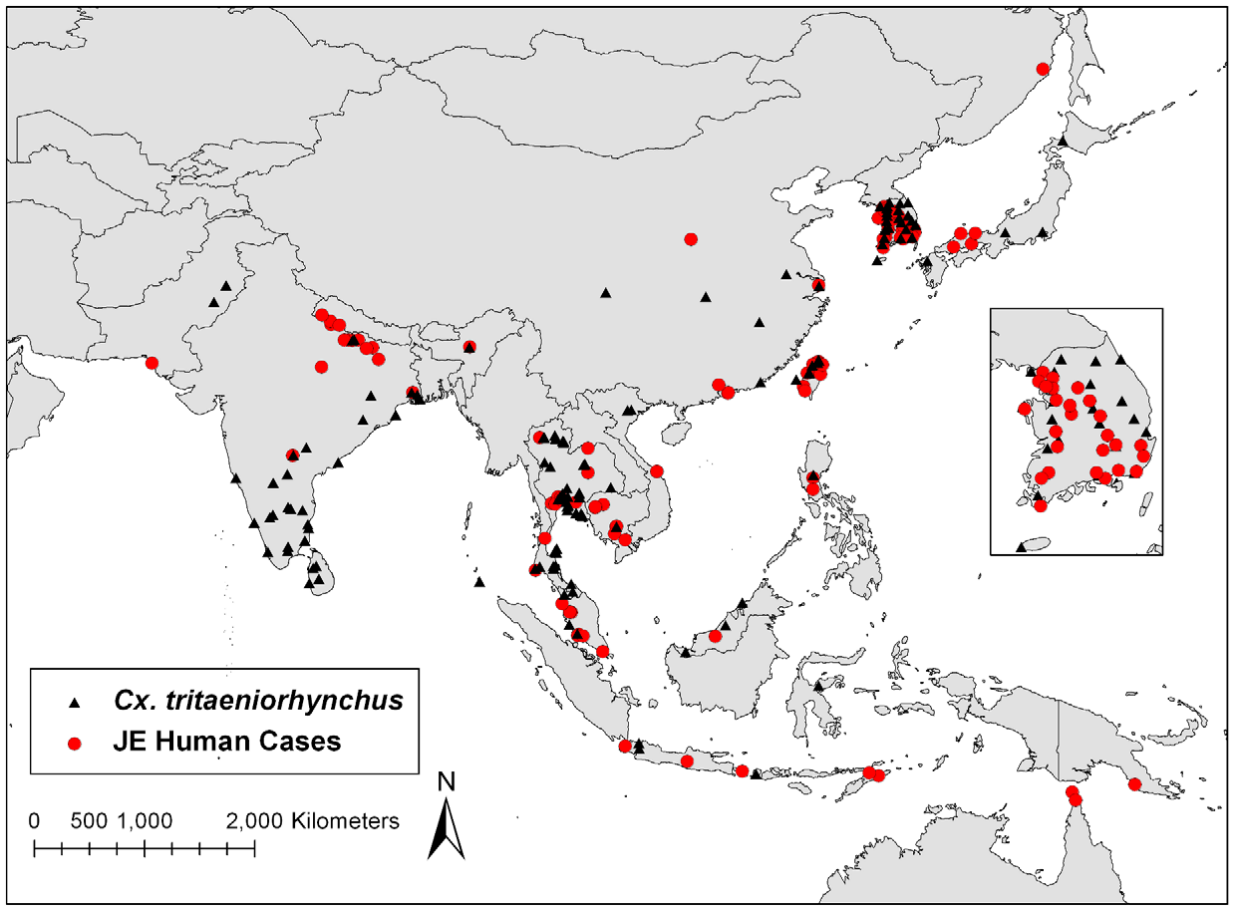
Distribution of known *Cx. tritaeniorhynchus* locations and documented human cases of JE within endemic region.

Approximate locations of known human JE cases were determined using locations provided in ProMED mail reports (www.promedmail.org) from 1994 through 2010 (Figure 2). Additional locations of confirmed JE cases were also determined through a PubMed literature search. Exact geographical coordinates were not reported for most documented human cases and were therefore extrapolated using the Google Earth software to obtain the latitude/longitude coordinates of the reported city, town, or village in which JE was documented.

### Vaccination Programs in JEV Endemic Countries

JEV vaccination program information was obtained from “Japanese Encephalitis Morbidity, Mortality and Disability: Reduction and Control by 2015” published in 2009 by the Program for Appropriate Technology in Health (PATH), Armed Forces Research Institute of Medical Sciences, and BIKEN [15]. Additionally, JEV vaccination programs information was also obtained from the WHO/IVB database [23]. Countries lacking a JEV vaccination program were identified using information in the above mentioned publications and confirmed with additional literature searches. A summary of these data are listed in Table 1.

**Table 1:** Summary of JEV vaccination programs in endemic countries and predicted percentage of land with greater than 25% estimated probability of *Cx. tritaeniorhynchus* presence based on ecological niche model.

JEV Endemic Countries	JE vaccination program status^1^	Percentage of area >25% estimated probability of *Cx. tritaeniorhynchus* presence
Australia	Administered in endemic areas	4.6
Bangladesh	No Current Immunization Program	56.8
Bhutan	No Current Immunization Program	0
Brunei	No Current Immunization Program	16.3
Cambodia	No Current Immunization Program	79.4
China	National Vaccination Program 2010	3.6
East Timor	No Current Immunization Program	68.4
India	Vaccine administered in high risk areas, not integrated into routine immunization program	19.2
Indonesia	No Current Immunization Program	14
Japan	National Vaccination Program 2010	42.4
Laos	No Current Immunization Program	19.7
Malaysia	Regional vaccination	8.4
Myanmar	No Current Immunization Program	20.5
Nepal	Vaccine introduced in 2006, not widely implemented	2.8
Democratic People's Republic of Korea	DPRK originated vaccine provided in high risk areas	21.1
Pakistan	No Current Immunization Program	0.06
Papua New Guinea	No Current Immunization Program	11.1
Philippines	No wide scale vaccination program in place , vaccine trial in progress	46
Republic of Korea	Government mandated mass immunization began in 1971	78.8
Russia	No data	0.01
Singapore	No data	10.7
Sri Lanka	18 of 26 districts receive vaccine annually, plans to extend to all districts	85.2
Taiwan	National vaccination program	34.2
Thailand	National Vaccination Program 2010	80.9
Viet Nam	Vaccine distributed in high risk districts	61
Western Pacific (Guam, Saipan)	No data	No data

1 Obtained from PATH: Japanese Encephalitis Morbidity, Mortality, and Disability: Reduction and Control by 2015 (ref.16) and WHO/IVB database, 193 WHO Member States. Data as of September 2011 (ref. 24).

### Environmental Data

One kilometer resolution climate and elevation data were obtained from WorldClim (http://www.worldclim.org/bioclim). The WorldClim organization has processed 50 years of ground-based weather measurements to produce mean monthly minimum and maximum temperatures and precipitation in a grid format at several different resolutions. The data were further processed to produce bioclimatic variables (e.g., mean temperatures of the wettest quarters). For this project, the highest resolution data available from WorldClim (approximately 1 km) were downloaded. In addition to bioclimatic variables, global elevation data obtained from WorldClim was re-sampled to 1-km resolution from NASA's Shuttle Radar Topography Mission (SRTM). Descriptions of the bioclimatic and elevation variables used for this study are listed in Table 2. To better understand the effect of each environmental variable on *Cx. tritaeniorhynchus* distribution, the values of each environmental layer at each site were extracted using ArcGIS (ESRI, Redlands, California, www.esri.com). This allowed for a comparison to the known environmental and distribution limitations for *Cx. tritaeniorhynchus* in the literature.

**Table 2:** Minimum, maximum, mean values and percent contribution of environmental data layers for *Cx. tritaeniorhynchus* model.

Variable1	Description	Min	Max	Mean	Percent Contribution
Alt	Altitude (elevation above sea level), m	0	838	153.4	9.6
Bio01	Annual mean temperature, °C	8.2	28.9	23.3	4.4
Bio02	Mean diurnal range (Mean of monthly (max temp-min temp)), °C	4.9	15	9.4	4.7
Bio03	Isothermality [(Bio2/Bio7)*100], °C	2.2	9	5.1	1.8
Bio04	Temperature Seasonality (standard deviation * 100), °C	30.1	1031.3	366	3.3
Bio05	Max temperature of the warmest month, °C	25.8	42.5	33.4	1.2
Bio06	Min temperature of the coldest month, °C	−12.5	24.4	12.4	3.3
Bio07	Temperature annual range (Bio5-Bio6), °C	7.2	40.7	21	3.2
Bio08	Mean temperature of the wettest quarter^1^, °C	16.9	32.8	262	21.7
Bio09	Mean temperature of the driest quarter, °C	−4.9	28.7	19.5	0.3
Bio10	Mean temperature of the warmest quarter, °C	20.1	34.3	27.7	0.6
Bio11	Mean temperature of the coldest quarter, °C	−4.9	27.2	18.3	1
Bio12	Annual precipitation, mm	152	4005	1610.3	16.2
Bio13	Precipitation of the wettest month, mm	41	1011	319.4	5.9
Bio14	Precipitation of the driest month, mm	0	233	30.8	6
Bio15	Precipitation seasonality (coefficient of variation), mm	18	138	74.5	5.8
Bio16	Precipitation of the wettest quarter, mm	95	2455	797.4	1
Bio17	Precipitation of the driest quarter, mm	0	786	114.9	0.7
Bio18	Precipitation of the warmest quarter, mm	62	1015	467.2	0.7
Bio19	Precipitation of the coldest quarter, mm	11	1812	260.7	8.5

1 A quarter is a period of three months.

A map of rice growing areas was created by processing GeoCover-LC (Land Cover) data from MDA Information Systems, Inc. (http://www.mdafederal.com/geocover/geocoverlc). GeoCover was created by processing Landsat Thematic Mapper images to create land cover maps for most areas of the world. Each pixel within the GeoCover-LC represents 30 by 30 meters. To convert the image to a resolution that could be used in the Maxent model, ArcGIS was used to count the number of rice pixels within each square kilometer (33 by 33 pixels). Then, a rice percentage was calculated for each square kilometer (number of rice pixels divided by total number of pixels in 1 km) and stored in a final output image.

### Ecological Niche Model

The Maxent 3.2.1 modeling program (http://www.cs.princeton.edu/~schapire/maxent/) was utilized to model the distribution of *Cx. tritaeniorhynchus* based on previously obtained geographical locations. Maxent utilizes a maximum entropy algorithm to analyze values of environmental layers, such as temperature, precipitation, and elevation, at known locations of species occurrence (collection records) to estimate the probable range of the species over a geographic region [22], [24]. This model is based on presence-only data instead of presence/absence data due to the lack of available absence data. Although absence data can be informative for modeling, ecological niche models based on presence-only data are useful in regions with limited collection data [22]. Without absence data, the true probability of presence cannot be modeled. In Maxent, which uses presence only data, the species distribution is output as an estimated probability map [25].

The Maxent program calculates the importance of environmental variables in developing predictive species distribution models by using the jackknife test of variable importance. The jackknife test runs the model 1) once with all variables, 2) dropping out each variable in turn, and 3) with a single variable at a time. Variables are considered import if they produce high training gains when used alone in a model. A variable is also important if the training gain is low when the variable is removed from the model [22].

Maxent utilizes two approaches to validate the accuracy of the model. The first method randomly selects occurrence points to be withheld from the model building to use as testing points. Using multiple definitions, a set of thresholds split the continuous probability values of the model into ‘predicted presence or absence’ categories. Maxent then calculates the p-value based on the null hypothesis that testing points will be predicted as “present” no better than by a random model. The second method calculates the Area Under the Curve (AUC) of the receiver operator characteristic (ROC), a graphical depiction of the sensitivity versus one (1) minus the specificity of the model often used to validate ecological niche models [22], [26]. The AUC indicates whether the model predicts species location better than a random distribution. AUC values of ≤0.5 indicates a random distribution and AUC values >0.9 indicates high reliability of the model [22]. To determine the best combination of environmental data for modeling, the model was run four times using different sets of input layers each time: 1) bioclimatic layers, elevation and rice crop data, 2) bioclimatic layers and elevation data, 3) bioclimatic layers only, and 4) elevation data only.

## Results

### Ecological Niche Model of *Cx. tritaeniorhynchus*

**Figure 3:**
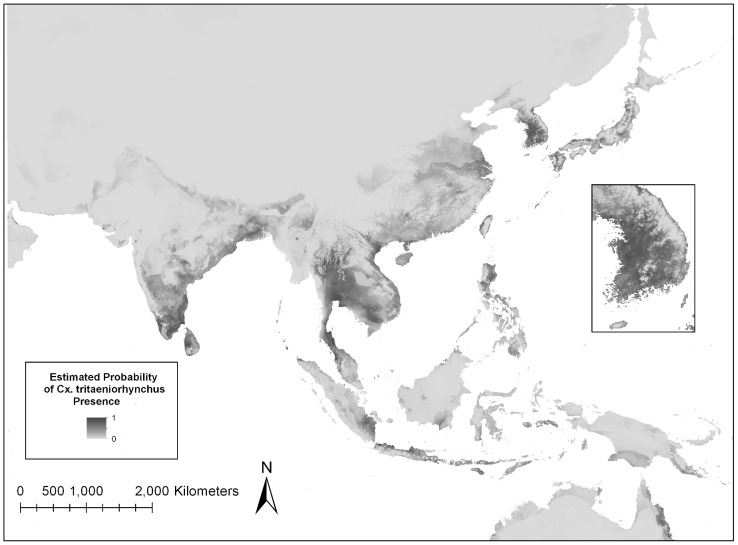
Maxent model estimation of the probability of *Cx. tritaeniorhynchus* distribution in the JE endemic region. Darker areas indicate areas that are likely to have suitable habitat for this vector species while lighter areas indicate areas of that are less suitable for the vector.

A total of 139 unique sites of documented *Cx. tritaeniorhynchus* geographical locations were utilized to construct the ecological niche model (Figure 3). Of the 139 total points, 105 (76%) were randomly designated as training points in order to build the model and 34 (24%) points were used to test the model. The model was run four times using different combinations of environmental layers (Table 3). Statistical results indicate that the most accurate model included bioclimatic layers and elevation (Table 3), and therefore this model was used in all subsequent analyses. Statistical evaluation showed the model to have a high accuracy, with the AUC>0.9 and low p-values. The model is available to view or download from www.vectormap.org.

**Table 3:** Maxent model accuracy analysis using different sets of environmental data inputs.

Environmental Data Input	AUC Training Points	AUC Test Points	P-value Minimum Training Presence
Bioclimatic and Elevation data	0.971	0.932	<0.0001
All layers (including rice crop)	0.968	0.919	<0.0001
Bioclimatic data only	0.968	0.929	<0.0001
Elevation data only	0.822	0.849	<0.0001

In order to evaluate the contribution of each environmental variable to the model, Maxent utilizes a jackknife test, which indicated that the annual precipitation (bio12) environmental layer is the environmental variable with the highest gain when used in the model by itself. The Maxent program also calculates a percent contribution for each variable in the model. The annual precipitation variable contributed 16.2% of the information used by the model, another indication that it is an important environmental factor for estimating the distribution of *Cx. tritaeniorhynchus* (Table 2). The mean temperature of the wettest quarter variable (bio08) contributed the highest percentage (21.7%) of the information to the model. Elevation was also an important variable, contributing 9.6% to the model. From the jackknife test, if elevation data were removed from the model, the overall training gain would decrease the most, indicating the elevation variable contained the most unique information of the variables in the *Cx. tritaeniorhynchus* distribution model.

The values of each environmental variable at each recorded location of occurrence were extracted using ArcGIS (Table 1). For example, the known locations for *Cx. tritaeniorhynchus* used in the model fell within 0 and 838 meters of elevation. This is consistent with the published reports that *Cx. tritaeniorhynchus* is rarely collected above 1,000 meters [27], [28].

### Human JE Cases and Vector Presence Estimation

**Figure 4:**
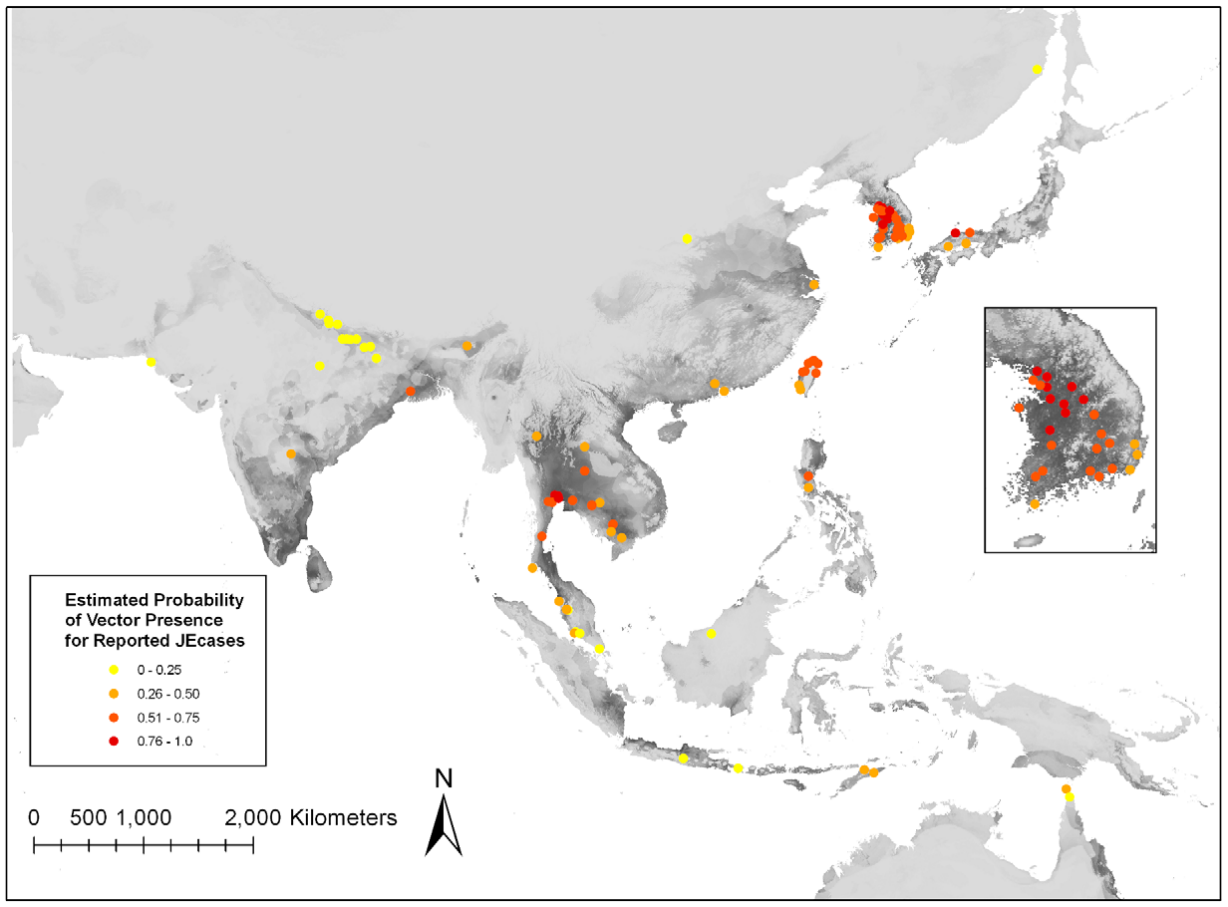
Human JE cases categorized by color based on the estimated probability of *Cx. tritaeniorhynchus* presence.

Ninety-six reported JE case locations were identified in endemic regions (Figure 2). ArcGIS analysis categorized human JE cases based on the estimated probability of *Cx. tritaeniorhynchus* presence (Figure 4). Human JE cases were identified at locations with a range of estimated probability of vector presence, including regions with 25% or less estimated probability. However, the majority (>75%) of human JE cases were reported from regions with greater than 25% estimated probability of *Cx. tritaeniorhynchus* presence. Limited availability of location data of human JE cases greatly impacts any associations between areas of high estimated vector probability and disease. For instance, the lack of human JE cases in other regions of estimated high probability of vector presence could be due to lack of reporting, improper diagnosis, or due to successful prevention strategies.

### *Cx. tritaeniorhynchus* Presence Estimation Per Country

ArcGIS analysis determined the approximate percentage of each country with >25% probability of *Cx. tritaeniorhynchus* presence based on the Maxent model (Table 1). Of the 25 endemic countries, seven possessed >50% of their land area with a higher probability of *Cx. tritaeniorhynchus* presence. Three countries (Bhutan, Pakistan, and Russia) possessed <1% of their total country area with a 25% probability of *Cx. tritaeniorhynchus* presence.

## Discussion

In this study, a statistically significant ecological niche model for *Cx. tritaeniorhynchus* was developed using mosquito presence records, climate, and elevation variables. Locations of human cases of JE generally fell within the higher probability areas of *Cx. tritaeniorhynchus* (Figure 4). Regions of estimated high probability of *Cx. tritaeniorhynchus* presence (Figure 3) are representative of preferred environments, based on temperature, precipitation and elevation where *Cx. tritaeniorhynchus* habitats occur. This model serves as a tool to fill in knowledge gaps regarding *Cx. tritaeniorhynchus* and can be utilized by health care professionals and policy officials in endemic regions to help guide the development and implementation of disease mitigating strategies in endemic regions.

The Maxent program identifies important environmental variables that are major contributors to the vector distribution model. Based on the jackknife test of variable importance, the annual precipitation (bio12) is an important contributor to the model. Additionally, the mean precipitation of the wettest quarters (bio08) and elevation also contributed greatly to the model for distribution of *Cx. tritaeniorhynchus* (Table 2). Previous studies that aimed to identify favorable ecological conditions of mosquitoes found that the optimal temperature of JEV vectors is between 22.8 and 34.5°C [29]. The importance of temperature during the wet season in the model is attributed to temperatures and flooded habitats that are optimal for larval development and adult survival. Locations in which the temperatures do not fall into the optimal range during the rainy season may therefore experience fewer mosquitoes, despite harboring the appropriate habitat. Temperature also plays a role in disease transmission rates, as higher temperatures increase the rates of virus replication and dissemination, while decreasing the time from mosquito infection to transmission of the virus to animal and human hosts [30].

Sampling bias is an issue that affects the accuracy of the model as the model was developed using existing data from the literature and VectorMap. Therefore, some regions have not been sampled in the study area and some have been oversampled. *Cx. tritaeniorhynchus* data for China were very limited (Figure 2), which may mean that some potential environmental conditions of *Cx. tritaeniorhynchus* were not represented in the model, in particular, the cooler Northeast region of China. Because modeling was limited to Cx. *tritaeniorhynchus*, there is a potential that for some regions, other primary or secondary vectors, i.e., *Cx. annulirostris*, *Cx. bitaeniorhynchus*, and *Cx. vishnui*, may predominate and maintain transmission of JEV in these areas. Collection records of *Cx. tritaeniorhynchus* were obtained spanning many decades and at different times during the year, furthering the impact of sampling bias on our model. In addition, the density of *Cx. tritaeniorhynchus* was not collected in this study and is an important limitation as vector abundance plays a crucial role in disease transmission. Further collection studies are therefore needed to determine the abundance of vector species in addition to presence in endemic regions.

**Figure 5:**
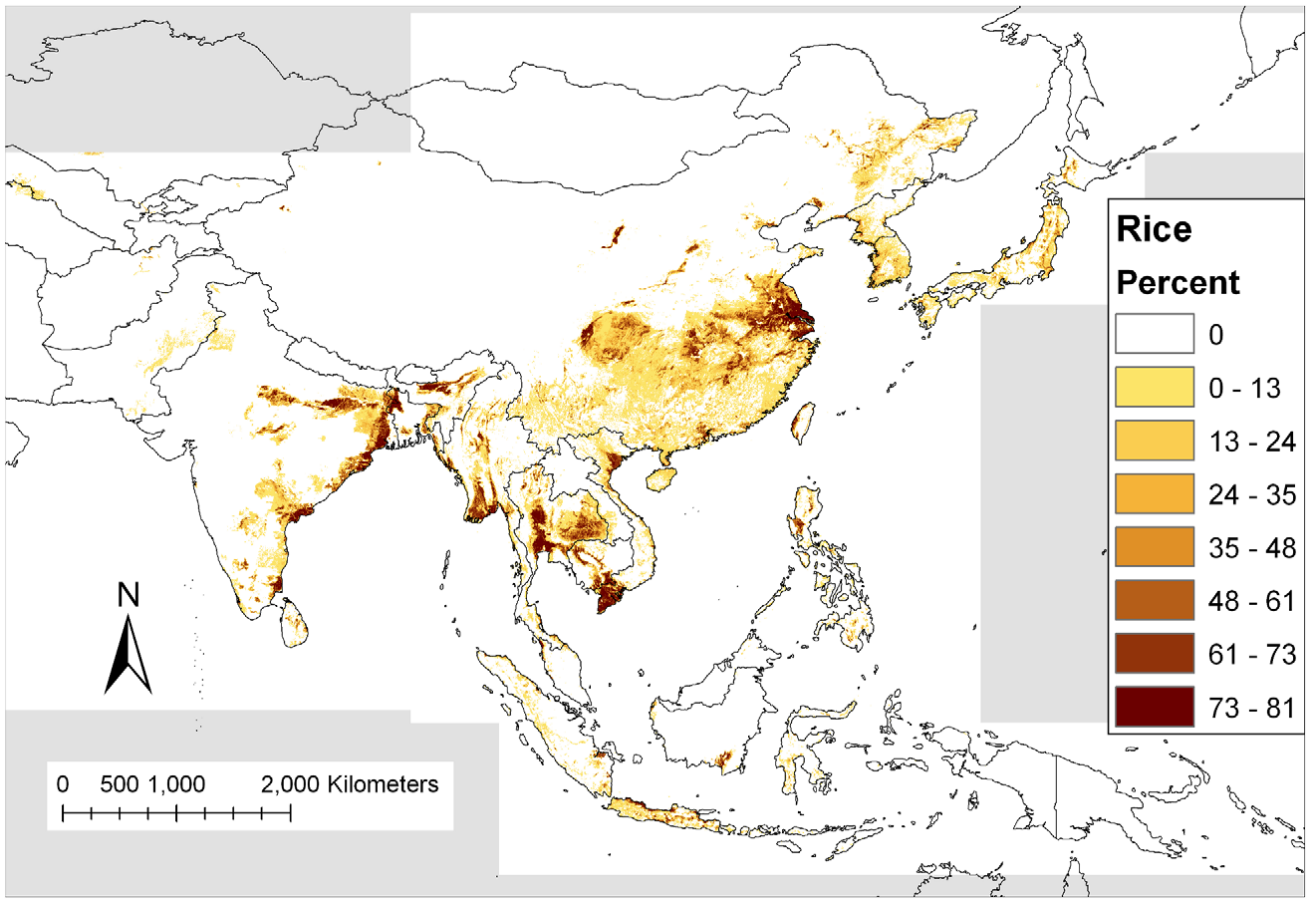
Percent of 30 meter pixels classified as rice land cover within 1 one square kilometer derived from the GeoCover Land Cover product. Gray areas indicate no data.

Low-lying flooded areas containing grasses, including rice paddies, are the primary larval habitats for *Cx. tritaeniorhynchus*. An increase in the amount of flooded rice field habitat has shown to be positively correlated with increases in adult populations of *Cx. tritaeniorhynchus* in the ROK [31]. Although the rice map derived from the GeoCover Land Cover map (Figure 5) does generally match the predicted occurrence of *Cx. tritaeniorhynchus*, there are some areas where the model predicts the presence of the mosquito, yet no rice crops were mapped. For some areas, rice may not have been identified correctly on the satellite images, since agricultural areas were limited or were adjacent to other predominant habitats. For example, rice is produced in Nepal [32], but no rice fields were identified by GeoCover in Nepal, since the identification of small rice fields in mountainous areas on satellite images can be difficult. Alternatively, this shows that environments other than rice fields are suitable habitat for *Cx. tritaeniorhynchus*.

The predicted probability of *Cx. tritaeniorhynchus* presence values were used to determine the percentage of a country at high risk (greater than 25% probability) for vector presence (Table 1). Many Asian countries have high percentages of their total land area with a >25% probability for the presence of *Cx. tritaeniorhynchus*. Cambodia, the ROK, Sri Lanka, and Thailand have over 75% of their land area with a >25% probability of *Cx. tritaeniorhynchus* presence. Countries demonstrating >50% of their total land area and with *Cx. tritaeniorhynchus* occurrence >25% probability includes: Bangladesh, East Timor, and Vietnam. However, some countries may have small areas of vector habitat close to large populations that can result in outbreaks despite low percentage estimated probability in the region overall. Further, this analysis does not take into account country size or vector abundance, which would also impact disease transmission. Although additional factors contribute to JE disease risks, the distribution of the vector populations within a country is a valuable data set when considering the necessity of vaccination and other health risk reduction programs.

Human JE cases were categorized based on the estimated probability of vector presence at the reported location (Figure 4). Interestingly, a portion of human cases were reported from regions with 25% or less estimated probability of *Cx. tritaeniorhynchus* presence. JE cases not falling within high probability pixels could have been acquired in nearby locations. Even for precisely located case data, the high resolution of the model (one kilometer pixels) increases the likelihood that the predicted *Cx. tritaeniorhynchus* location does not match disease acquisition location as many people travel more than a kilometer in the course of a typical day. Alternatively, additional factors other than the presence of *Cx. tritaeniorhynchus* may be important when determining the risk of disease. For instance, other JEV vectors may dominant in these regions estimated with low *Cx. tritaeniorhynchus* presence. JE is one of many febrile illnesses that affect human populations in Asia. Difficulties arise in diagnosis of JE in patients based on symptoms alone that range from mild to very severe, with laboratory tests required for confirmation. Obtaining geographical data of where human cases were acquired is made difficult due to lack of confident diagnoses, patient travel history, and spatial data. The lack of precision of the reported case locations may also contribute to lower numbers of JE cases falling within high probability *Cx. tritaeniorhynchus* pixels. Identification of human JE cases in this study is extremely limited and does not represent all human cases in JE endemic regions. In many cases, only a village or city name was given for reported cases. A previous study to model the distribution of *Cx. tritaeniorhynchus* to predict JE in the Republic of Korea found human cases to occur in areas of high estimated probability of vector presence [20]. This study, however, utilized intensive vector collection methods and JE case data were obtained from the Korea Centers for Disease Control resulting in an overall more extensive and accurate model. This illustrates the need for increased surveillance of vector and human JE cases in order to generate more accurate risk models for JE. In order to evaluate the impact of vector presence on the risk of JE in humans, comprehensive efforts to identify specific locations of both symptomatic and asymptomatic JE cases across endemic regions are needed.

Ecological niche modeling inherently possesses limitations in that it makes predictions based solely on environmental variables that impact larval development and adult survival. Other important factors that influence vector distributions include: vector control strategies, public health campaigns, socioeconomic status, human population densities, anthropogenic changes to land (creation of vector habitat), vector species competition, and predator influences on their potential distribution and population densities. Further, the use of WorldClim data may underestimate or ignore environmental variables that occur during a short time period or transient habitat suitable for the vector to survive. Incorporation of these variables will undoubtedly increase the validity of the model. These factors are also important to take into consideration when implementing mosquito control initiatives and vaccination campaigns.

The reemergence of JEV remains possible due to multiple factors. Increases in the pig farming industry, modification and expansion of arable lands for wetland rice farming, and a fraction of the population unvaccinated/non-immune, in combination with optimal climatic conditions, contribute to the potential for periodic outbreaks of JE as the one observed in the ROK [9]. Genotype analyses of circulating JEV strains identified the reemergence of genotype V, which was unseen in Asia for over 50 years [33], [34]. The identification of emerging/reemerging JEV strains is important for vaccine development and the implementation of effective vaccination programs. Increased surveillance in areas with known vector populations and additional risk factors, such as reservoir and amplifying hosts, will aid in the identification of circulating JEV strains as well as strains that are emerging in novel human and vector populations. Understanding the vector distribution is a key step to effectively understanding JEV risks and also to preventing additional outbreaks of JE in endemic countries.
